# Draft Genome Sequences of Hazelnut-Associated *Pseudomonas* Strains Isolated in Ontario, Canada

**DOI:** 10.1128/MRA.01021-20

**Published:** 2020-11-05

**Authors:** Walid Ellouze, Darlene Nesbitt, Michael Parcey, Steven Gayder, Geneviève Marchand, Antonet M. Svircev, James T. Tambong

**Affiliations:** a Agriculture and Agri-Food Canada, Vineland Station, Ontario, Canada; b Centre for Biotechnology, Brock University, Saint Catharines, Ontario, Canada; c Agriculture and Agri-Food Canada, Harrow, Ontario, Canada; d Agriculture and Agri-Food Canada, Ottawa, Ontario, Canada; University of Arizona

## Abstract

Two *Pseudomonas* strains (H346-M and H346-S) were isolated from hazelnut trees showing symptoms of shoot dieback and necrosis. The draft genome sequences of H346-M and H346-S consist of 66 and 51 contigs, respectively, with total sizes of 5,693,988 and 5,889,925 bp and 4,885 and 5,045 protein-coding sequences, respectively.

## ANNOUNCEMENT

Shoots and leaves showing symptoms of necrosis and dieback, typical of bacterial blight, were collected from European hazelnut trees (*Corylus avellana* L. cv. Jefferson) at the experimental farm of the Agriculture and Agri-Food Canada Research and Development Centre in Harrow, Ontario, Canada. The leaves and shoots were surface sterilized for 1 min in 1% sodium hypochlorite and rinsed three times with sterile water. Three to five sections of symptomatic tissues were excised to expose the margin between healthy and diseased tissues, added to 0.5 ml sterile water, macerated with a pipette tip, and incubated for 5 min at room temperature. Fifty microliters of the supernatant was plated onto nutrient agar (Difco, MD, USA) and incubated at 27°C for 48 h. Twenty-one distinct colonies were isolated in pure culture and stored on Microbank cryobeads (Pro-Lab Diagnostics, ON, Canada) at −80°C. Of the 21 isolates, H346-M and H346-S were identified as *Pseudomonas* using 16S rRNA sequencing (1) and BLAST analysis (2). One cryobead was spread across a nutrient agar plate for each isolate and incubated at 27°C for 48 h. A single colony of each isolate was suspended in 50 μl of 0.01 M phosphate buffer (pH 6.8), and 4 μl was used as the template for PCR (1).

Genomic DNA extraction of H346-M and H346-S from 12-h-old cultures of one cryobead incubated at 27°C in 1 ml Luria-Bertani broth (Difco) was performed using the bacterial genomic DNA isolation kit (Norgen Biotek Corp., ON, Canada). Libraries were constructed using the NEBNext Ultra II DNA library prep kit for Illumina, and raw reads were generated by paired‐end Illumina MiSeq sequencing (Génome-Québec, Montreal, Canada). Totals of 460,722 and 541,872 paired-end reads (each 250 bp long), totaling 115,180,500 and 135,468,000 bp, were generated for strains H346-M and H346-S, respectively. The quality of the reads was checked using FastQC v0.11.9 (3). *De novo* assemblies were done using Unicycler v0.4.8 (4), a SPAdes optimizer, as implemented in Pathosystems Resource Integration Center (PATRIC) (5), and contigs with a length of <300 bp were discarded. The draft genome sequence of strain H346-M contains 66 contigs (minimum, 365 bp; maximum, 570,275 bp; *N*_50_, 174,852 bp; total size, 5,693,988 bp), while that of strain H346-S has 51 contigs (minimum, 318 bp; maximum, 915,726 bp; *N_50_*, 399,281 bp; total size, 5,889,925 bp). The G+C contents are 59.29% and 59.36%, and the estimated coverages are 19× and 23× for H346-M and H346-S, respectively. Default parameters were used for all software unless otherwise specified.

Based on PATRIC (5), the two draft genome sequences have 100% completeness and 94% fine consistency. The NCBI Prokaryotic Genome Annotation Pipeline (PGAP) (6) annotated the draft genome sequence of strain H346-M to have 4,885 protein-coding sequences, 57 tRNAs, and 3 rRNAs (1 5S rRNA, 1 16S rRNA, and 1 23S rRNA). Strain H346-S has 5,045 protein-coding sequences, 53 tRNAs, and 4 rRNAs (2 5S rRNAs, 1 16S rRNA, and 1 23S rRNA). Also, each strain has 4 noncoding RNAs. Strains H346-M and H346-S share 3,607 PATRIC cross-genus protein families (PGfams) (5), with 449 and 321 unique PGfams, respectively. Genome-based average nucleotide identity (7) and *in silico* DNA-DNA hybridization (8) analyses taxonomically identified strain H346-M as a putative Pseudomonas congelans strain, while H346-S is a new strain of Pseudomonas cerasi.

### Data availability.

This whole-genome shotgun project was deposited in DDBJ/ENA/GenBank under accession numbers JACSZR000000000 and JACSZQ000000000 for H346-M and H346-S, respectively. The versions described here are the first, JACSZR010000000 and JACSZQ010000000. The raw reads were deposited in the NCBI SRA under accession numbers PRJNA659801 and PRJNA659798.
